# The Role of MMP8 in Cancer: A Systematic Review

**DOI:** 10.3390/ijms20184506

**Published:** 2019-09-11

**Authors:** Krista Juurikka, Georgina S. Butler, Tuula Salo, Pia Nyberg, Pirjo Åström

**Affiliations:** 1 Cancer and Translational Medicine Research Unit, Faculty of Medicine, University of Oulu, 90014 Oulu, Finland; krista.juurikka@oulu.fi (K.J.); pia.nyberg@oulu.fi (P.N.); pirjo.astrom@oulu.fi (P.Å.); 2 Medical Research Center Oulu, Oulu University Hospital and University of Oulu, 90220 Oulu, Finland; 3 Department of Oral Biological & Medical Sciences, Faculty of Dentistry, University of British Columbia, Vancouver, BC V6T 1Z2, Canada; george.butler@ubc.ca; 4 Centre for Blood Research, University of British Columbia, Vancouver, BC V6T 1Z3, Canada; 5 Department of Oral and Maxillofacial Diseases, Faculty of Medicine, University of Helsinki, 00014 Helsinki, Finland; 6 Helsinki University Hospital, 00014 Helsinki, Finland; 7 Translational Immunology Research Program (TRIMM), University of Helsinki, 00014 Helsinki, Finland; 8 Biobank Borealis of Northern Finland, Oulu University Hospital, 90220 Oulu, Finland

**Keywords:** matrix metalloproteinase 8, cancer, prognosis, molecular mechanism, cancer drug therapy, systematic review

## Abstract

Matrix metalloproteinases (MMPs) have traditionally been considered as tumor promoting enzymes as they degrade extracellular matrix components, thus increasing the invasion of cancer cells. It has become evident, however, that MMPs can also cleave and alter the function of various non-matrix bioactive molecules, leading to both tumor promoting and suppressive effects. We applied systematic review guidelines to study MMP8 in cancer including the use of MMP8 as a prognostic factor or as a target/anti-target in cancer treatment, and its molecular mechanisms. A total of 171 articles met the inclusion criteria. The collective evidence reveals that in breast, skin and oral tongue cancer, MMP8 inhibits cancer cell invasion and proliferation, and protects patients from metastasis via cleavage of non-structural substrates. Conversely, in liver and gastric cancers, high levels of MMP8 worsen the prognosis. Expression and genetic alterations of MMP8 can be used as a prognostic factor by examination of the tumor and serum/plasma. We conclude, that MMP8 has differing effects on cancers depending on their tissue of origin. The use of MMP8 as a prognostic factor alone, or with other factors, seems to have potential. The molecular mechanisms of MMP8 in cancer further emphasize its role as an important regulator of bioactive molecules.

## 1. Introduction

The number of new cancer cases is expected to grow in the future as the lifetime risk of developing cancer rises with increasing aging of the population [1]. Survival has increased in many cancers [2] due to improvements in prevention [3], detection [4], diagnosis and treatment [5]. These advances have been facilitated by extensive basic and clinical research utilizing in vitro methods, genetic analysis and cell culture methods, as well as in vivo animal studies and clinical trials. Moreover, the identification of an increasing number of cancer biomarkers has improved cancer diagnostics and care [6]. Biomarkers, such as DNA, RNA, proteins, peptides or chemical modifications of biomolecules, are used to assess the risk of developing a specific cancer, and to measure treatment responses and cancer progression [7]. Historically, biomarkers have been explored in blood, urine and tumors using immunoassays and immunohistochemistry.

Members of the matrix metalloproteinase (MMP) family of zinc-dependent proteinases have been proposed as biomarkers and therapeutic targets for various cancers [8]. These proteinases have been mostly considered tumor-promoting because of their indisputable role in one of the hallmarks of cancer progression—the degradation of the extracellular matrix (ECM) that correlates with cancer cell invasion and metastasis [9]. MMP8, also known as neutrophil collagenase or collagenase-2, cleaves triple helical type I collagen and numerous other ECM and non-ECM substrates [10]. MMP8 is released from polymorphonuclear neutrophils (PMN) and hence plays an important part in mediating inflammation. For example, expression of MMP8 is increased in gingivitis as well as periodontitis [11] and MMP8 has been shown to suppress neuroinflammation [12] and inflammation in osteoarthritis [13]. Although mostly released from neutrophils, MMP8 is also expressed by various other cells [14]. However, the functions of MMP8 seem to be highly diverse, and thus, no clear consensus for its role in cancer has been reached. Moreover, its molecular mechanisms in different cancer types are still largely unknown.

The previous review articles on the role of MMP8 in cancer are outdated, do not focus solely on MMP8 and are not systematic reviews on the subject [15,16]. By applying systematic review guidelines and methods, we gathered the original research articles published on MMP8 related to cancer and provide a comprehensive compilation of the current knowledge on the subject. We aim to create an overview of the potential prognostic value of MMP8 in blood and tumor samples as well as highlight the effect of genetic changes in MMP8 for prognosis. In vitro and in vivo (murine) studies are reviewed to emphasize the molecular mechanisms of MMP8 and finally, the use of MMP8 in all levels of cancer therapy is discussed.

## 2. Results

### 2.1. The Potential Use of MMP8 for Evaluating Cancer Prognosis

Prognosis refers to the likely course, duration and outcome of a disease based on its characteristics, such as tumor size in cancer. The statistically calculated terms of overall survival (OS), disease-free survival (DFS) and recurrence-free survival (RFS) are often used to describe the prognosis of a patient. The Dictionary of the National Cancer Institute defines these terms as the average time from the start of treatment to when the patient is still alive (OS), or the time from the end of treatment for which the patient survives without any signs or symptoms of that cancer (DFS, sometimes referred as recurrence- or relapse-free survival, RFS). Prognostic biomarkers are biomolecules, such as DNA or protein, which inform the clinician of the patient’s status or enable them to predict the patient’s prospects, e.g., chance of recurrence [7]. Here we evaluated the measurement of MMP8 (gene expression and protein levels) in tumors and circulation, as well as genetic polymorphisms, as a prognostic marker.

#### 2.1.1. Analysis of Tumoral MMP8 on Protein and mRNA Level

Levels of different molecules have been traditionally studied by visualizing target antigens using immunohistochemical methods on fresh frozen or formalin-fixed, paraffin-embedded (FFPE) tumor samples [17]. Tumor lysates can also be used as a source of protein for analysis [18]. Sometimes the tumor samples are collected as microarrays (tumor microarray, TMA) [19]. Tumoral mRNA expression has been usually studied by reverse transcriptase-polymerase chain reaction (RT-PCR) using RNA extracted from fresh or FFPE tumor tissue [20]. Yet mRNA can also be visualized in tumor sections by other methods such as in situ hybridization [21]. The studies on tumoral MMP8, both mRNA and protein, are collected in Table 1.

##### 2.1.1.1. No Clear Evidence for the Use of MMP8 Protein as a Prognostic Factor in Breast Cancer

MMP8 has been widely studied in breast tumors. Many studies have found no correlation between tumoral MMP8 expression and clinicopathological parameters [25,28,29]. Köhrmann et al. [26] detected a correlation between tumor grade and MMP8 protein levels, yet Sarper et al. [62] noticed a loss of MMP8 in myoepithelial cells during carcinogenesis. Gutierrez-Fernandez et al. [27] showed that MMP8 gene expression negatively correlated with lymph node involvement, but so far no other study has confirmed the correlation. Another study on breast cancer showed an association between MMP8 levels and the levels of metalloproteinase inhibitor 1 (TIMP-1) and MMP9 but not with tissue plasminogen activator, urokinase or their inhibitor (PAI-1) [30,63].

##### 2.1.1.2. The Prognostic Value of Tumoral MMP8 Protein Levels in Skin Cancer Depends on the Subtype

A higher level of MMP8 in the epidermal-dermal border correlates with melanoma invasiveness [57]. However, in basal cell carcinoma, MMP8 protein was only detected in the dermis and no correlation to collagenolytic activity was observed [58]. Similarly, in squamous cell carcinoma (SCC) of the skin, the epithelial level of MMP8 was not useful in distinguishing chronic, non-malignant leg ulcers from SCC [56], nor to explain the aggressiveness of SCC in organ transplant recipients [64]. However, MMP8 was slightly more frequently detected in neutrophils in keratoacanthomas (~44%) compared to SCCs (~33%) [55].

##### 2.1.1.3. Tumoral MMP8 Protein Level is an Applicable Biomarker Only in Tongue SCC among All Head and Neck SCCs

Analysis of MMP8, performed in a variety of different head and neck SCCs (HNSCCs), localized MMP8 protein to tumor cell islands, PMNs, plasma cells and fibroblasts [47]. MMP8 is abundant in oral squamous cell carcinoma (OSCC) tumors, but the higher level does not correlate with clinicopathological features nor does it increase the survival of the patients [40,41]. Additionally, MMP8 was shown to be present in the OSCC tumor interstitial fluid in samples collected during surgery and analyzed by mass spectrometry [65]. Yet in the SCC of oral (mobile) tongue (OTSCC), higher MMP8 in tumor favored better prognosis and lower mortality [44] although very high and negative levels hinted towards shorter DFS [42]. Furthermore, high vascular endothelial growth factor C (VEGF-C) together with low MMP8 levels in OTSCC tumors predicted worse prognosis even better than either of these factors separately [39]. Patients with tonsillar SCC showed higher MMP8 levels compared to patients with benign tonsillar disease [66]. MMP8 protein did not have a prognostic value in larynx tumors [43] where its levels varied greatly between patients, nor in supraglottic laryngeal tumors [45] where it was detected in only a few cells. Likewise, no difference in MMP8 protein levels were found between salivary gland carcinomas and normal tissue [46].

##### 2.1.1.4. Tumoral MMP8 Protein Level Associated with Malignancy in Ovarian and Liver Cancer and Variates in Colorectal and Gastric Cancer

Qin et al. [48] found that MMP8 levels positively correlated with transforming growth factor β1 (TGF-β1) levels in hepatocellular carcinoma samples and their reciprocal increase was correlated with cancer stage, metastasis and shorter time-to-recurrence. High cytoplasmic levels of MMP8 in ovarian cancer cells correlated to tumor stage and overall poor prognosis, although not strongly enough to be useful as a prognostic marker [50]. In the fluid of ovarian cysts, elevated levels of MMP8 was associated with tumor malignancy [51]. It can be concluded, that high MMP8 seems to have a tumor-predicting role in both liver and ovarian cancers. In colorectal cancer, correlation between the step-wise increase of MMP8 levels and tumor malignancy was found [33]. Yet, others [31,34] did not find such a correlation and Väyrynen et al. found the MMP8 protein mostly in necrotic areas and neutrophils [32]. Similarly for gastric cancer, MMP8 level was higher in well-differentiated tumors, yet it did not affect patient survival [38]. In contrast, Laitinen et al. [35] found that negative MMP8 staining was associated with stage I cancer, T1 tumor stage as well as no lymph node metastasis and furthermore, women with negative MMP8 staining had a better prognosis.

For the following cancers, only single studies on the role of MMP8 exist. In the case of lung cancers, MMP8 level, and possibly also its effect, depend on the cancer subtype [49]. Pancreatic adenocarcinomas showed higher MMP8 levels compared to normal tissue [53], especially in patients with short survival time [52]. In uterine cancer, MMP8 was more abundant in tumor samples compared to normal tissue, but its level was not linked to any clinicopathological features such as tumor grade [61]. In chondrosarcoma [24] and soft tissue neoplasms [59], MMP8 staining was either absent or weak, and thus, no conclusions could be made of its role. In jaw cysts, MMP8 was only detected in plasma cells [67], and in osteosarcoma it was detected in about 50% of biopsy and resection samples but not at all in metastases [23].

##### 2.1.1.5. MMP8 mRNA Expression is Rarely Detected in Patient Samples

**Figure 1:**
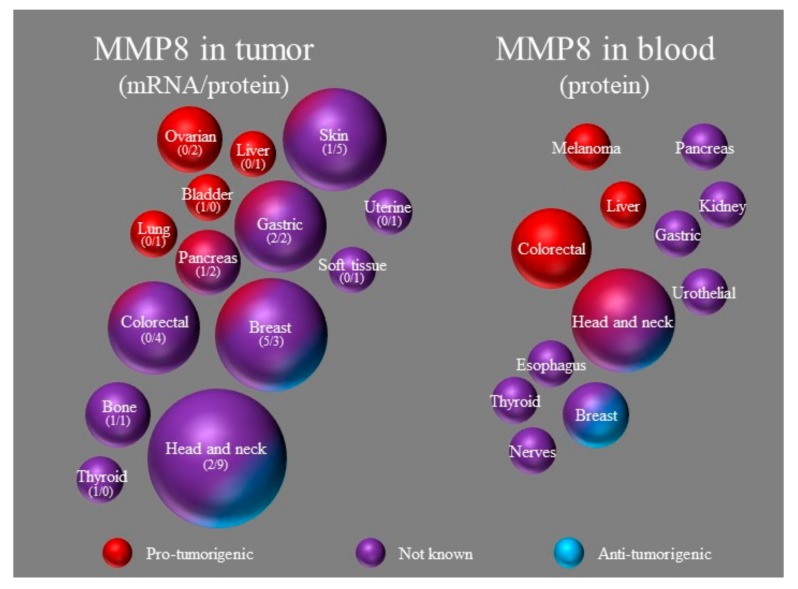
The effect of MMP8 in tumor or blood on cancer prognosis. The size of the ball is in portion to the number of publications on that cancer, the number in brackets depicts studies on mRNA or protein. The color of the ball depicts the effects of MMP8 on that cancer.

The expression of MMP8 mRNA has been detected only in a few chondrosarcomas [24], hepatocellular carcinoma [68] and gastric cancer samples [37]. In bladder tumors, MMP8 mRNA expression was not strong enough to show significant correlation to malignancy [22]. MMP8 mRNA was elevated in skin samples of basal cell carcinoma patients compared to healthy controls [54] and in malignant thyroid neoplasms compared to benign neoplasms [60]. Similarly, Merkerova et al. [69] and Bruchova et al. [70] both noticed an up-regulation of MMP8 gene expression in the blood leukocytes of chronic myeloid leukemia patients compared to healthy controls. Additionally, they showed that MMP8 expression was not connected to hydroxyurea treatment and it can be effectively silenced in vitro, but the effects of this expression were not discussed. On the other hand, MMP8 mRNA expression was lower in gastric adenocarcinoma tumors compared to paired healthy tissue [36]. It could be that MMP8 mRNA is not stable or that the protein expression detected in tumors does not originate from cancer cells. The effects of tumoral MMP8 on cancer prognosis are illustrated in Figure 1 along with the studies on MMP8 in blood.

#### 2.1.2. MMP8 Levels in the Serum or Plasma

The circulatory system refers to the organ systems that circulate blood and lymph throughout the body—both great sources of disease biomarkers. The studies on MMP8 in the circulation of cancer patients have predominantly investigated MMP8 in blood, specifically in plasma and serum. Linkov et al. [71] revealed that MMP8 levels in serum are unique to each individual, but they are also stable, indicating that especially if the baseline level of the patient was known, MMP8 could be used as a prognostic marker. No similar studies have been performed on plasma samples. Table 2 collates the studies on circulatory MMP8 in various cancers.

##### 2.1.2.1. MMP8 Levels Increase with Malignancy in HNSCC Patients

Kuropkat et al. [79,80] found higher MMP8 levels in the serum of HNSCC patients compared to healthy patients and MMP8 levels correlated with the T- and N-status and overall TNM stage (TNM classification of malignant tumors, Union for International Cancer Control’s[UICC]). Plasma MMP8 levels did not correlate with survival or lymph node involvement [78]. Further analysis showed that high MMP8 levels in plasma correlated with tumor stage IV and showed a trend (statistically not significant) towards favorable outcomes [77].

##### 2.1.2.2. High Serum MMP8 Level in Digestive System Cancers Predict Worse Prognosis

High serum MMP8 levels seem to hinder the survival of patients with various cancers of the digestive and urinary systems. In colorectal cancer patients, increased serum MMP8 levels were measured compared to healthy controls and a high MMP8 level correlated with malignancy, reduced survival and increased systemic inflammation [32,74,75]. Moreover, in hepatocellular carcinoma, low serum MMP8 levels correlated with better overall survival [82]. In pancreatic and kidney cancer patients, serum MMP8 levels were increased compared to healthy controls but no correlation to any clinicopathological features was found [81,85]. Interestingly, low or high MMP8 serum levels correlated with poor prognosis in gastric cancer patients [35]. In regard to esophageal cancer, MMP8 levels were significantly lower in serum samples from early stage patients compared to healthy controls [76]. MMP8 levels in plasma showed no correlation to clinical stage or cancer-related mortality in bladder cancer patients [87].

##### 2.1.2.3. The Prognostic Value of Circulating MMP8 Levels in Other Cancers Requires More Studies

In breast cancer patients, serum MMP8 levels were higher compared to healthy controls [72]. MMP8 expression in plasma was higher in patients with non-inflammatory breast cancer as well as in patients with lymph node involvement, but interestingly, lower in patients with risk of distant metastasis [73]. Similarly, higher plasma MMP8 levels were demonstrated in carotid body paraganglioma patients compared to healthy persons, but not patients with malignant disease [84]. Low serum MMP8 levels in melanoma patients correlated with better overall survival [83]. Serum MMP8 levels did not correlate with the presence of tumors in thyroid cancer patients [86]. High MMP8 expression, measured only from circulating tumor cells, in the blood of metastatic castration-resistant prostate cancer patients correlated with lower overall survival [88]. In lung cancer, measuring MMP8 levels in bronchial washings had no value in terms of detection [89].

#### 2.1.3. Genetics of MMP8 in Cancer

**Figure 2:**
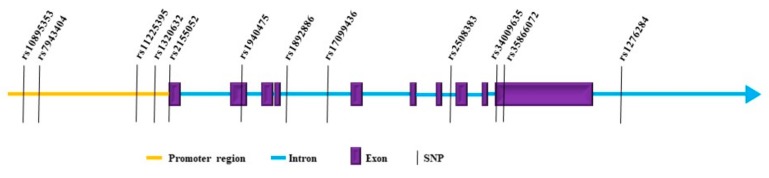
Schematic illustration of MMP8 including the studied single nucleotide polymorphisms (SNPs).

Cancer is a genetic disease in origin and various mutations, polymorphisms and DNA copy number variances have been detected in different cancers. Studies on MMP8 genetics in cancer have focused largely on single nucleotide polymorphisms (SNPs), which are collected in Table 3. Figure 2 illustrates the locations of studied SNPs in relation to the MMP8 gene.

##### 2.1.3.1. The SNP rs1122539 Protects from Breast and Bladder Cancer but Increases the Risk of Melanoma and Ovarian Cancer

The most studied SNP in the MMP8 gene is SNP rs11225395 (C-799T) located in the promoter region that increases the transcription of MMP8 [98]. SNP rs11225395 is associated with a lower risk of developing breast cancer and a higher overall survival from breast cancer as shown by three independent studies [95,96,98]. Yet Hsiao et al. [94] found no correlation with breast cancer risk in their population. Another study found that the same SNP correlated with reduced risk of bladder cancer, even in smokers, but was not associated with tumor grade [91].

In contrast, SNP rs11225395 was associated with an increased risk of developing melanoma [96] and ovarian cancer. Moreover, in ovarian cancer, a tendency towards worse overall survival was found [107]. In the case of HNSCC, no correlation between SNP rs11225395 and survival of the patients was found [78], nor was this SNP associated with risk of oral cancer [100]. Studies on the susceptibility to hepatocellular carcinoma [103], bladder cancer [90], acute lymphatic leukemia in children [102] and lung cancer [104] revealed no correlation to SNP rs11225395.

##### 2.1.3.2. Fluctuating Findings on the Effect of SNP rs1940475

The SNP rs1940475 (A259G) is located in the pro-domain of MMP8 and alters the structural stability of the domain, thus decreasing the activation of MMP8 [110]. Murugan et al. [109] revealed that 73.6–88.8% of their thyroid carcinoma tumor samples harbored SNP rs1940475 and the authors postulated, based on the location of this SNP, an effect on the function of MMP8. Later Lin et al. [36] showed decreased enzymatic activity of MMP8 harboring this SNP as well as increased recurrence and lower survival of gastric adenocarcinoma patients. Yet Kader et al. [92,93] found no correlation with the risk of bladder cancer or its type to SNP rs1940475. Interestingly though, this SNP had a seemingly protective effect in former smokers, but in never smokers there was a trend of increased risk of invasive bladder cancer. Nan et al. [111] associated SNP rs1940475 with the risk of basal cell carcinoma (but not SCC or melanoma) but the association was not significant in age-adjusted analysis. Based on the studies, these cancers might benefit from having a MMP8-inactivating SNP suggesting that MMP8 is a tumor-protective factor in them.

##### 2.1.3.3. Other MMP8 SNPs Also Decrease Cancer Risks

Gonzalez-Arriaga et al. [105] found that SNP rs2155052 (C17G) in the MMP8 promoter region is associated with decreased risk of lung cancer, especially in high-risk patients such as ever-smokers. An intronic SNP rs1892886 is associated with breast cancer [97] and was also found to have a lower frequency in patients with lymph node metastasis [98], along with SNPs rs11225395, rs1940475 and rs1276284. On the contrary to other studies, SNP rs17099462 decreased the expression of MMP8 and was shown to be associated with the increased risk of death from ovarian cancer [106]. MMP8 SNPs rs35866072 and rs34009635 have no effect on the risks of childhood acute lymphocytic leukemia [102], breast [94], bladder [90], oral [100] or lung cancer [104]. No MMP8 SNPs were correlated to nasopharyngeal carcinoma [101] or gastric cancer and its clinicopathological features [99].

##### 2.1.3.4. Studies on Somatic and Epigenetic Changes Strengthen the View of Active MMP8 as a Tumor-Suppressive Factor

Only a few genetic studies on MMP8 other than SNP analyses have been reported. Palavalli et al. [112] found that melanomas harbor somatic MMP8 mutations, which decrease MMP8 activity and lead to increased colony formation and cell migration in vitro as well as metastasis formation in vivo. Accordingly, a microarray analysis of the epigenetic regulation of MMPs in breast cancer and glioma cell lines revealed an epigenetic inactivation of MMP8 unlike other MMPs, which could explain the reduction of active MMP8 in various malignancies unrelated to their genetics [113]. For miRNA studies, miR-539 was shown to directly target MMP8 3′-UTR along with other targets thus inhibiting MMP8 expression. In osteosarcoma cells, the overexpression of this miRNA led to reduced proliferation, invasion and migration, suggesting MMP8 acts as a tumor-promoting factor in osteosarcoma [114].

### 2.2. In Vitro Experimental Evidence and In Vivo Mouse Studies Elucidate the Molecular Mechanisms of MMP8 in Cancers

#### 2.2.1. Studies in Skin Cancer Paved the Way for the Idea of Tumor-Suppressive MMP8

In vitro studies on skin cancer revealed that most melanoma cell lines, but not normal melanocytes, express MMP8 mRNA [57]. Yet at the protein level, MMP8 was only present in half of the nine melanoma cell lines examined by Giricz et al. [115]. Similarly, MMP8 mRNA expression in keratinocytes of varying tumorigenic potential was detected in vitro but protein expression varied [116].

In vivo murine studies paint a clearer picture; male MMP8 knock-out (KO) mice were more prone to carcinogen-induced skin and connective tissue cancers than the wild-type mice and the protective effect of MMP8 could be restored by a bone marrow transplant from wild-type mice [117]. Subsequently, MMP8 KO mice were also shown to be susceptible to melanoma [27]. Murine melanoma cells, B16F10, formed more lung metastases in MMP8 KO mice, whereas the overexpression of MMP8 in tumor cells lowered the number and size of metastases. MMP8 was also upregulated in the secretome of DNA-dependent protein kinase, catalytic subunit (DNA-PKcs)-silenced melanoma cells that metastasized less in vivo and migrated/invaded less in vitro [118], providing indirect evidence for the protective role of MMP8. However, in B16F10-derived melanoma in C-C chemokine receptor 5 KO mice, MMP8 had no effect on metastasis [119].

#### 2.2.2. MMP8 Has Fluctuating Expression Profiles in Breast Cancer Cells In Vitro, but Rather Consistent Tumor-Protective Effects In Vivo

Overall MMP levels increase during malignant transformation in breast cancer cell lines—so does MMP8 as well [120]. Most studies [121,122,123], apart from Agarwal et al. [124], show that the non-metastatic MCF-7 cell line produces no endogenous MMP8 protein. Yet, notoriously metastatic MDA-MB-435, as well as less metastatic MDA-MB-468 [26,121] and MDA-MB-231 cells [122], do produce MMP8. MMP8 mRNA expression is only detected in MDA-MB-231 cells [121,123,124], except in one study that found no expression [125]. Co-culture with adipose tissue-derived mesenchymal stem cells, a driver of tumorigenesis, did not affect the MMP8 expression of breast cancer cells [126]. Although MMP8 was mainly found to be expressed by metastatic cell lines and not the non-metastatic MCF-7 cell line, many [123,124,127] have shown that MMP8-expressing breast cancer cell lines are less invasive in vitro and generate less metastases in mice in vivo. Similar conclusions were drawn by Decock et al. [128] and Soria-Valles et al. [129] in their studies on MMP8 KO mice. Systemic MMP8 expression was found to decrease tumor size, hinder tumorigenesis and protect from metastasis formation. Moreover, xenografted tumors in MMP8-deficient mice showed decreased vascularization and neutrophil recruitment [128]. In their studies, MMP8 cleaved decorin, which inhibits TGF-β1: The subsequent downregulation of miR-21 expression led to activation of known tumor suppressors such as programmed cell death 4 (PDCD4) [129]. MMP8 has also been shown to affect the protease web by, e.g., decreasing the levels of MMP3 [128] and MMP9 [62] in breast cancer cells, which was speculated to decrease the malignant behavior of the cells [128]. Furthermore, MMP8 overexpression was shown to increase the expression of interleukins 6 and 8 in breast cancer cells [123], which was postulated to be connected to inflammatory response rather than cancer progression. Accordingly, MMP8 overexpressing breast cancer cells had reduced migratory and proliferative properties in vitro [129]. Similarly, MMP8 overexpressing breast myoepithelial cells show increased adhesion, due to retraction fiber shortening and differential localization of integrin α6β4 [62]. The variable results might be explained by heterogeneity between cell lines, sensitivity differences between methods, or as shown by Bachmeier et al. [121]; confluence of the cell culture. However, MMP8 was shown not to be involved in the tumor-promoting effects of TLR9 and relaxin that induced the activity of many MMPs in breast cancer cells thereby increasing cell invasiveness [130,131]. In contrast, tumor-induced systemic inflammation in breast cancer increased metastasis generation in mice due to neutrophil-mediated tumor cell extravasation and inhibition of tumor cell clearance. Additionally, these neutrophils were shown to secrete increased amounts of MMP8 and MMP9, which facilitated tumor cell extravasation and metastasis [132]. Thus, MMP8 expression, although low in non-malignant breast cancer cells, seems to act as a tumor suppressor rather than an enhancer in malignant cells but not in neutrophils. It is important to note that factors elevated during cancer progression should not be interpreted as tumor promoters.

#### 2.2.3. Tumor-Protective Molecular Mechanisms of MMP8

**Figure 3:**
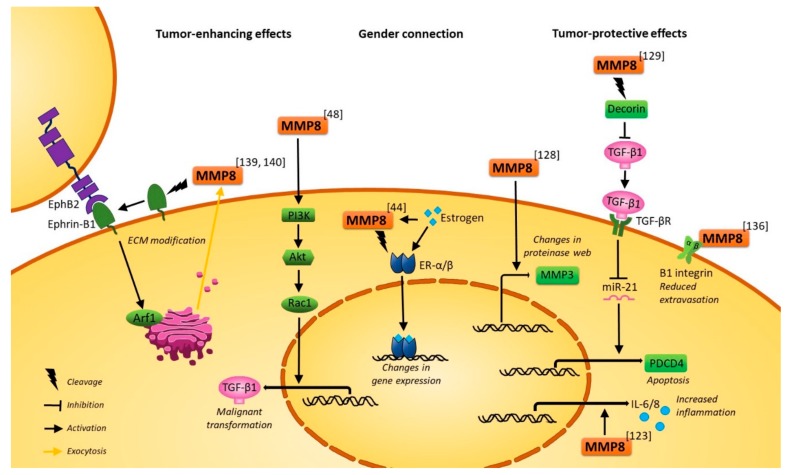
Suggested molecular mechanisms of MMP8. Abbreviations: Akt: RAC-α serine/threonine-protein kinase; Arf1: ADP-ribosylation factor 1; ECM: Extracellular matrix; EphB2: Ephrin type B receptor 2; ER-α/β: Estrogen receptor α/β; IL-6/8: Interleukin 6/8; MMP3: Matrix metalloproteinase 3; MMP8: Matrix metalloproteinase 8; PDCD4: Programmed cell death 4; PI3K: Phosphoinositide 3-kinase; TGF-βR: Transforming growth factor β receptor: TGF-β1: Transforming growth factor β-1. See references (in superscript) for original publications.

Several studies show that MMP8 triggers tumor-suppressive molecular cascades after cleavage of various non-ECM substrates, as shown in Figure 3. Indeed, it has also been shown that collagenolytic activity did not decrease in fibroblasts isolated from MMP8 KO mice [133], suggesting other MMPs than MMP8 were the source of collagenolysis in vivo. However, Korpi et al. [44] found that female MMP8 KO mice are especially prone to carcinogen-induced tongue cancer and that MMP8 cleaves estrogen receptors α and β. Later, MMP8 overexpression was shown to reduce the invasion and migration of OTSCC cells in vitro and to change the gene and protein expression of various factors, including a decrease in VEGF-C [39]. Lung adenocarcinoma cells treated with hepatocyte growth factor variants showed inhibition of proliferation, reduced invasion and increased amounts of active MMP8, which was suggested to facilitate the tumor-protective effects [134]. On the other hand, MMP8 mRNA expression increased along other MMPs in aggressive lung cancer cell lines resistant to targeted chemotherapy [135]. Additionally, Pellinen et al. [136] showed that silencing of MMP8 in prostate cancer cells led to increased ligand binding of β1 integrin and higher prostate cancer cell invasion in vitro as well as increased breast cancer lung extravasation in vivo. Yet overexpression of integrin α6β4 in OSCC cells did not change MMP8 mRNA expression, which shows, that MMP8 affects integrin but not vice versa [137].

#### 2.2.4. Tumor-Promoting Molecular Mechanisms of MMP8

MMP8 exhibits some pro-tumorigenic effects through substrate cleavage: Ephrin-B1 belongs to an ephrin family of proteins, which transduce signals related to cell adhesion and angiogenesis [138]. The intracellular C-terminus of ephrin-B1, that is required for invasion of pancreatic cancer cells in vitro and in vivo in mice, was shown to regulate the release of MMP8 from these cells via activation of the cell trafficking regulator, Arf1 [139]. Furthermore, the released MMP8 could cleave the ectodomain of ephrin-B1, in a potential feedback mechanism to downregulate the activity of ephrin-B1. Later it was shown that a peptide from the ephrin-B1 C-terminus can block the release of MMP8 and can inhibit the invasion of gastric scirrhous carcinoma cells in vitro and their peritoneal dissemination in vivo in mice [140]. Although MMP8 in vitro was shown to cleave laminin5 γ2-chains (a basal membrane component known to induce migration when cleaved), its addition to MCF-7 breast cancer cells had no impact on their migration over laminin [141].

Other pro-tumorigenic effects of MMP8 may be linked to signaling properties of MMP8: MMP8 itself upregulates TGF-β1 expression by activating the PI3K/Akt/Rac1-pathway, which in turn leads to an epithelial-mesenchymal transition (EMT), and subsequently, increased invasion and migration of hepatocellular carcinoma cells [48]. Bone marrow cells produce MMP8 along with other MMPs during multiple myeloma progression in vivo and the tumor growth can be reduced with the broad spectrum MMP inhibitor; SC-964 [142]. Various factors known to promote tumorigenesis were shown to increase MMP8 expression or activity in vitro: CD44- and CD24-expressing renal cell carcinoma stem cell subsets, serpinA1-expressing gastric cancer cells and CCL25-expressing ovarian cancer cells all showed higher migratory and invasive potential in vitro as well as higher MMP8 expression [143,144,145]. Interestingly, in prostate cancer cells, no MMP8 was detected even after induction with CCL25 [146]. In vitro in OSCC cells, betel quid increased MMP8 levels and cell migration [147], whereas trypsin-2 activated MMP8 [148]. Yet, endostatin, anti-angiogenic collagen XVIII fragment had no inhibitory effect on MMP8 although it could inhibit migration [149]. However, none of these studies examined how changes in the level of MMP8 affected the cancer cells.

#### 2.2.5. MMP8 Expression Is Not Detected at All in Some Cancer Cell Lines

In some cancer cell lines, no MMP8 mRNA was detected, such as in bladder cancer [150], Ewing’s sarcoma [151], chondrosarcoma [152] or follicular thyroid carcinoma cells [153], and thus, the mechanistic role of MMP8 in those cancers has not been examined. Additionally, no MMP8 gene expression was found in stellate cells—building blocks of the ECM in pancreatic cancer [154]. In renal cell carcinoma, no differences in MMP8 gene expression were detected between healthy and cancerous kidney tissue as well as bone metastasis [155]. MMP8 expression is detected in fibrosarcoma cell lines, but not in the in vivo chorioallantoic membrane (CAM) assay, which is used to study tumor angiogenesis and invasion [156].

### 2.3. The Potential of MMP8 in Cancer Treatment

#### 2.3.1. Using MMP8 as a Pharmaceutical Adjuvant Shows Promising Results

Three independent studies show that MMP8 reduces interstitial tumor fluid pressure, increases fluid flow in various murine tumors, namely lung cancer, soft tissue sarcoma and HNSCC, and enhances the efficacies of oncolytic virus therapy and liposomal drugs [157,158,159]. Thus, MMP8 could be a potential adjuvant drug. Additionally, variating levels of MMPs, MMP8 included, can be visualized with tomography methods in breast cancer tumors in vivo (murine), offering a diagnostic tool to evaluate tumor progression and heterogeneity [160].

#### 2.3.2. Cancer Treatments Affect MMP8 Levels in Tumors

Many cancer drugs can decrease MMP8 levels along with other MMPs: The plant-based isoflavones genistein and glycitein, as well as the flavone apigenin, down-regulate MMP8 expression in vitro and inhibit leukemia cell invasion [161,162] and decrease the viability of hepatocellular carcinoma cells [163]. Saponins from the plant Rhizoma paridis decreased the expression of inflammatory cytokines and MMP8 as well as reduced lung cancer tumor volume and growth in mice [164]. Therapeutic gold nanoparticles, which decreased ovarian cancer tumor growth and metastasis in vivo, also inhibited ovarian cancer cell proliferation, decreased MMP8 expression, and altered cytokine production in vitro [165]. Treatment with the cytotoxic peptide HNP-1 reduced the levels of MMP8 as well as increased lactate dehydrogenase toxicity in OSCC cell lines but did not affect cell viability [166]. Reduced levels of MMP8 were observed when glioblastoma multiforme spheroids were treated with 5-aminolevulinic acid-photodynamic therapy [167]. MMP8 levels in blood decreased after tumor removal in paraganglioma [168] and pancreatic ductal adenocarcinoma patients [85]. Systemic changes in MMP8 levels after cancer treatment might be explained by the changes in systemic inflammation.

On the other hand, several studies show that some cancer treatments may also increase MMP8 levels: Increased expression of proangiogenic factors, including MMP8, by myeloid derived suppressor cells could contribute to resistance to Sunitinib treatment seen in some kidney cancer patients [169]. Treatment of bladder cancer with bacterial CBM588 strongly inhibited the tumor growth in vivo in mice, which was suggested to be due to the release of TNF-related apoptosis-inducing ligand (TRAIL) from PMN cells by MMP8 [170]. An in vitro prostate cancer study by Reel et al. [171] showed that Zoledronate, an inhibitor of osteoclastic bone resorption, downregulates discoidin domain receptor proteins—known MMP activators. Subsequently, the MMP levels were downregulated except for MMP8, which increased. The progesterone receptor modulator CDB-2914, induces MMP expression, including MMP8, in uterine leiomyoma cells, but MMP8 is not activated by this treatment and its role remains unclear [172]. In skin cancer, immunosuppression does not change MMP8 expression levels or its location [173], nor did MMP8 expression in skin SCC tumors correlate with its aggressiveness in immunosuppressed transplant patients [64].

#### 2.3.3. High MMP8 Levels Can Guide Treatment Choices and Indicate Harmful Post-Operative Reactions in Some Patients

High serum MMP8 levels could be used to define cutaneous melanoma patients who would benefit from interferon-α (IFN-α) therapy [174]. Yet Van Roy et al. [175] showed earlier that MMP8 deficiency does not change the survival of mice inoculated with B16BL6 melanoma tumor cells and treated with TNF/IFNγ therapy. Lin et al. [176] showed that prostate cancer patients with MMP8 SNP rs11225395 benefit more from androgen deprivation therapy than other patients.

MMP8 levels in some body fluids can also be used to assess post-operative reactions: HNSCC patients, who developed radiochemotherapy-induced oral mucositis, had a higher salivary MMP8 level than those who did not develop this reaction [177]. Elevated MMP8 levels in intraperitoneal fluid were also seen in rectal cancer patients who developed anastomotic leakage after surgery [178].

#### 2.3.4. MMP8 as an Anti-Target for MMP Inhibitors

Inhibition of MMPs has been considered as a potential clinical intervention in cancer treatment. The first synthetic MMP inhibitors (MMPIs); Batimastat (BB-94) and Marimastat (BB-2516), were developed almost 20 years ago and they showed promising results in murine studies, e.g., by reducing the tumor volume in ovarian cancer [179]. They were rushed to clinical trials and disappointment ensued—no effects on tumors were seen, yet the side effects for patients, such as pain in bones, were significant [180]. Recently, the development of MMPIs has moved away from general MMPIs [181,182,183] towards specific inhibitors for MMP1 [184], gelatinases [185,186,187], MMP11 [188] and specific cancers [189] or stages of tumor progression [190]. However, specificity is an issue due to the high level of sequence and structural homology between MMPs, and some of these “specific” inhibitors also bind MMP8. Previously, inhibitors were designed specifically for MMP8 [191]. Since the tumor-protective effects were demonstrated in 2003, MMP8 has been regarded as an anti-target in MMP inhibitor development [192,193,194]. However, MMP8 expression has also been shown to increase in highly vascularized tissues such as tumor and placenta [195] and this protease is generally regarded as a proangiogenic factor. Angiogenesis is a major target in cancer treatment and MMPIs have been developed against proangiogenic MMPs such as MMP8 and MMP9 [108,196].

## 3. Discussion

In this systematic review we gathered the available data from the Scopus database on the role of MMP8 in cancer, including the use of MMP8 as a biomarker for prognosis in tumors, blood, and at the genetic level, in vivo and in vitro studies on its molecular mechanisms as well as its use in cancer therapy. The limitation of the study is the use of one database only. In addition, given the large number of studies included an in-depth analysis of individual studies was prevented. Meta-analyses were not performed due to the high variability between different studies as well as the poor number of studies per cancer type.

In general, MMP8 levels in tumors variate and are not always detected. The studies published to date, suggest that high MMP8 protein levels might predict better survival for tongue [39,44] and some breast [27] cancer patients, but they provide a worse prognosis in hepatocellular [48] and ovarian [50,51] cancers. Results variate greatly for some, such as colorectal cancer [31,32,33,34]. However, mRNA levels are even more inconsistent. This phenomenon can partly be explained by different sources of MMP8 in the body, such as tumor cells and neutrophils. Not all studies state where the analyzed MMP8 staining is localized (tumor cells, neutrophils, tumor stroma, etc.), which might partly explain the inconsistency. The presence of cancer seems to increase the level of MMP8 in circulation and depending on the cancer, high plasma MMP8 levels might be beneficial (e.g., breast cancer [73]), but in some cancers (colorectal [32,74,75], hepatocellular [82] and melanoma [83]) high serum MMP8 levels correlate with poor prognosis. Additionally, it should be noted that serum and plasma may give contradicting results; plasma contains blood cells including neutrophils, which might release additional MMP8 in the sample after collection. SNPs, which modulate MMP8 transcription and activity, seem to play the strongest role in cancer prognosis and are beneficial for breast [95,98], bladder [91,93] and gastric [36] cancer patients but detrimental in ovarian [107] cancer patients. Other SNPs and genetic modifications have been poorly studied or do not correlate with clinicopathological parameters.

In vitro experiments and in vivo murine studies shed light on the non-collagenolytic actions of MMP8 and strengthen the findings from in vivo patient data. These studies were the first to introduce the idea of tumor-protective MMP8 in skin cancer [27,117] and breast cancer [124,127,128,129]. In these cancers, MMP8 slows the metastatic process both in vivo and in vitro, which explains why patients benefit from MMP8. Studies have also revealed non-collagen substrates for MMP8 including transmembrane proteins, cytokines and signaling molecules. However, MMP8 activity can also be harmful, as shown for pancreatic and gastric cancers [138,140], in which the interaction between MMP8 and Ephrin-B1 seems crucial, and hepatocellular carcinoma [48], where MMP8 was shown to activate the PI3K/Akt/Rac1 pathway.

MMP8 is nowadays considered an anti-target in cancer drug development. However, many cancer therapies modulate MMP8 levels—this might present changes in systemic inflammation. The use of MMP8 in treatment selection and for the evaluation of harmful post-operative reactions has not been fully examined in various cancers but it might be useful in those cancers where MMP8 has been shown to play a role. The use of MMP8 as an adjuvant [157,158,159] in cancer drug treatment poses unappreciated potential and should be further studied.

The role of MMP8 seems more definite in those cancers, that are well studied—e.g., the effects of MMP8 in breast cancer is studied in more than 30 research articles. When the tumor-protective role of MMP8—a novel function for proteases at that time, was first reported in 2003 in breast and skin cancers, it probably increased the interest in the role of MMP8 specifically in those cancers. However, to evaluate the overall function of MMP8 in various steps of cancer progression, the tumor-promoting mechanisms of this multifunctional enzyme should also be studied.

## 4. Materials and Methods

**Figure 4:**
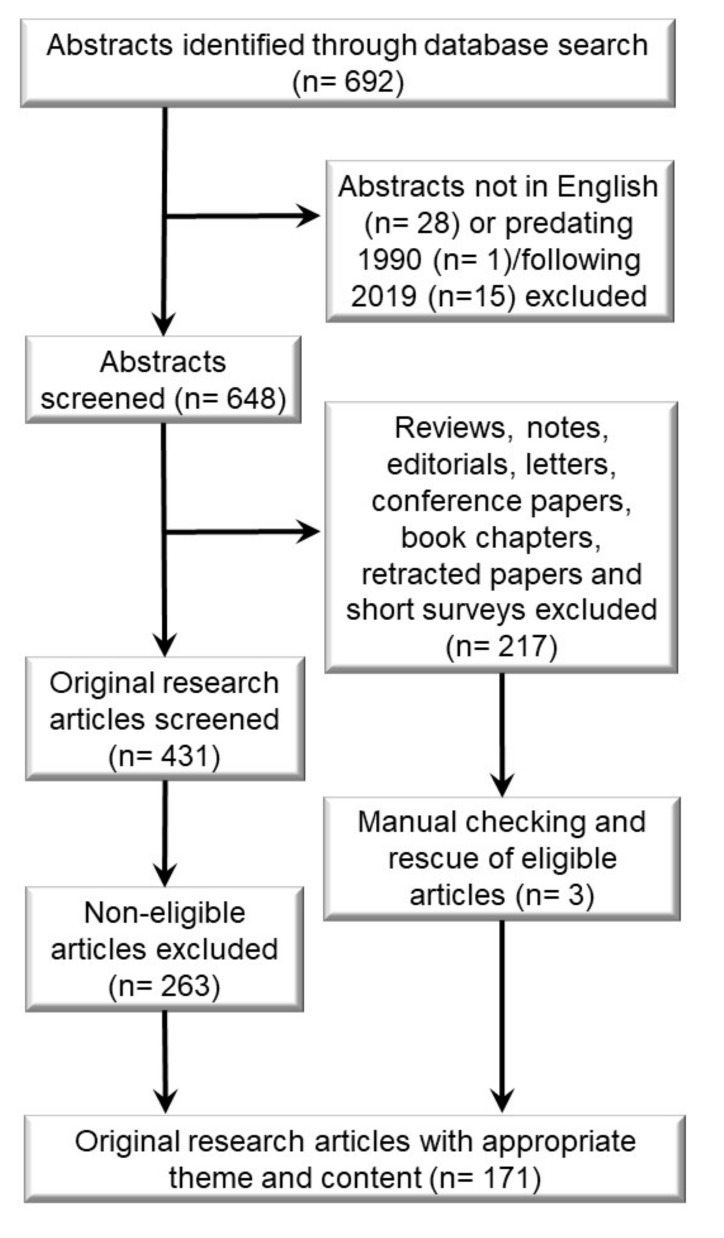
Workflow for literature search.

This article was compiled by following the systematic review guide—Preferred Reporting Items for Systematic Reviews and Meta-Analyses (PRISMA) [197]. The workflow is depicted in Figure 4. Searches were run using the Scopus (https://www.scopus.com) database with the assistance of an information specialist. The search terms MMP8 (mmp 8 OR matrix metalloproteinase 8 OR neutrophil collagenase OR collagenase 2) and cancer (cancer OR neoplasm* OR carcinoma* OR malignan* OR tumo?r* OR sarcoma* OR leukemi* OR lymphoma* OR adenocarcinoma*) were searched from titles, abstracts and keywords, where the asterisk (*) is used to indicate truncation and the question mark (?) is used to indicate wildcard characters.

A total of 692 hits were retrieved. Next, articles written in languages other than English, published before 1st January, 1990 and after 1st January, 2019 or which were not original research articles were excluded. After the first exclusion, the abstracts of the remaining 431 articles were carefully analyzed. After an abstract review, articles were excluded if they were a review, or if MMP8 was not studied, or if the study did not focus on cancer—leading to 168 collected articles. The articles excluded by Scopus (217) were manually checked and three articles were returned. Thus, a total of 171 articles were included in this systematic review. To answer our specific research questions (“can MMP8 be used as a prognostic factor in cancer?”; “what are the molecular mechanisms of MMP8 in cancer?”; and “can MMP8 be used in cancer treatment?”) the information about study size and type, methodology, patient outcome, main findings and statistical results were retrieved.

## Figures and Tables

**Table 1:** Tumoral matrix metalloproteinase 8 expression and prognosis.

Cancer	Focus	Method	Study Size (Patients + Healthy Controls)	Expression and Prognosis (*p*-Value)	Authors
Bladder	mRNA	RT-PCR	113 + 20	Positively correlates with tumor grade (*p* < 0.001).	Wallard et al. 2006 [22]
Bone	Protein	IHC	25 + 0	5/10 resection samples and 11/22 biopsies but 0/3 metastases show MMP8 staining.	Korpi et al. 2011 [23]
	mRNA	RT-PCR	29 + 0	5/29 chondrosarcoma tumors show expression.	Scully et al. 1999 [24]
Breast	mRNA	RT-PCR	39 + 16	Trend to positive correlation with tumor grade (ns).	Benson et al. 2013 [25]
	Protein, mRNA	RT-PCR, WB, IHC	20 + 5	No difference in mRNA. Protein correlates with stage (*p* ≤ 0.05).	Köhrmann et al. 2009 [26]
	Protein, mRNA	IHC, RT-PCR	280 IHC, 250 RT-PCR + 10	mRNA expression negatively correlates with LN involvement (*p* = 0.006). Better survival of patients without adjuvant therapy (*p* = 0.009).	Gutierrez-Fernandez et al. 2008 [27]
	mRNA	Microarray	295 + 0	No correlation to clinicopathological features.	McGowan & Duffy 2008 [28]
	Luminal A; mRNA	RT-PCR	25 + 0	No correlation to clinicopathological features.	Decock et al. 2007 [29]
	Protein	ELISA	55 + 0	No correlation to tumor size or LN metastasis.	Duffy et al. 1995 [30]
Colorectal	Protein	IHC	548 + 0	No correlation with clinicopathological features or prognosis.	Koskensalo et al. 2012 [31]
	Protein	IHC	5 + 0	Very little staining (0.1–5%) in cancer cells.	Väyrynen et al. 2012 [32]
	Protein	ELISA	100 + 0	Correlation with malignancy (*p* = NR).	Verspaget et al. 1999 [33]
	Protein	IHC	121 + 0	No expression in cancer cells.	Takeha et al. 1997 [34]
Gastric	Protein	IHC	276 + 0	Negative staining associates with stage I cancer (*p* = 0.022), T1 tumor (*p* = 0.005), diffuse type (*p* < 0.001), no LN metastasis (*p* = 0.016) and age under 67 (*p* = 0.007). Better prognosis for women with negative staining (*p* = 0.026).	Laitinen et al. 2018 [35]
	mRNA	RT-PCR	34 + 34	Lower mRNA expression in cancer tissue compared to paired healthy tissue.	Lin et al. 2017 [36]
	mRNA	RT-PCR	17 + 22	No difference between patients and controls and no correlation to clinicopathological features.	de la Pena et al. 2014 [37]
	Protein	ELISA	81 + 0	Expression higher (*p* ≤ 0.001) esp. in well-differentiated (*p* ≤ 0.002) tumors. No correlation to survival.	Kubben et al. 2006 [38]
Head and neck	OTSCC; protein	IHC	57 + 0	High VEGF-C (*p* = 0.001) and low MMP8 level (*p* = 0.01) correlate with shorter CSS, combined VEGF-C+/MMP8- status correlate with poor CSS (*p* < 0.001). No correlation to clinicopathological variables.	Åström et al. 2017 [39]
	OSCC, CSCC; protein	IHC	36 OSCC, 25 CSCC + 0	No correlation to clinicopathological features or overall survival.	Ahmed Haji Omar et al. 2015 [40]
	OSCC; protein	IHC	25 + 0	5/25 tumors showed moderate levels.	Lawal et al. 2015 [41]
	OTSCC: protein	IHC	70 + 0	No correlation to clinicopathological features.	Mäkinen et al. 2012 [42]
	Larynx; protein	AG array	7 + 5	Levels higher compared to normal mucosa (*p* = NR).	Korampalli et al. 2011 [43]
	OTSCC; protein	IHC	90 + 0	Correlation to better prognosis and lower-case fatality (*p* < 0.05).	Korpi et al. 2008 [44]
	SL, SCC; protein, mRNA	IHC, RT-PCR	32 + 32	<20% tumors showed expression (mRNA/protein).	Xie et al. 2004 [45]
	SG; protein	EIA	23 + 23	No differences to normal tissue.	Kayano et al. 2004 [46]
	SCC; protein, mRNA	IHC, ISH	19 + 0	Low mRNA expression and protein levels in all samples.	Moilanen et al. 2002 [47]
Liver	Protein	IHC	73 + 0	Co-overexpression with TGF-β1 predicts poor prognosis in HCC patients (*p* < 0.025). Correlation to cancer stage (*p* = 0.038) and metastasis (*p* = 0.049).	Qin et al. 2016 [48]
Lung	Protein	FACS	22 SCC, 19 AC + 0	Higher in tumor (*p* = 0.0084) and SCC vs AC (*p* = 0.0023). Trend towards positive correlation with recurrence (ns).	Shah et al. 2010 [49]
Ovarian	Protein	IHC	302 + 0	Correlation to tumor stage (*p* ≤ 0.01), grade (*p* ≤ 0.01) and poor prognosis (*p* = 0.019).	Stadlmann et al. 2003 [50]
	Protein	NR	NR	High MMP8 levels in ovarian cyst fluid are associated with malignancy (*p* = NR).	Stenman et al. 2003 [51]
Pancreas	Protein	LC-MS	9 SS, 10 LS + 0	MMP8 upregulated in SS group (*p* < 0.05).	Hu et al. 2018 [52]
	Protein, mRNA	IHC, RT-PCR	45 + 10	Expression higher (*p* = 0.04) in tumor versus healthy tissue. No correlation to clinicopathological features or survival. No mRNA found.	Jones et al. 2004 [53]
Skin	nBCC; protein, mRNA	RT-PCR, WB	22 + 22	Higher levels in nBCC tumors (*p* < 0.0001), but no differences in mRNA expression.	Ciążyńska et al. 2018 [54]
	SCC; protein	IHC	31 KA, 15 SCC + 0	More frequent levels in keratoacanthomas compared to SCCs (ns)	Kuivanen et al. 2006 [55]
	SCC; protein	IHC	9 SCC, 31 leg ulcers + 0	Cannot distinguish between non-malignant leg ulcers and SCC.	Impola et al. 2005 [56]
	Melanoma; protein	IHC	10 + 0	Correlates with invasiveness (*p* = NR).	Giambernardi et al. 2001 [57]
	BCC; protein	IHC	54 +16	No correlation to collagenolytic activity.	Varani et al. 2000 [58]
Soft tissue	Protein	ICC	39 + 0	6/39 tumors have MMP8 staining.	Roebuck et al. 2005 [59]
Thyroid	mRNA	cDNA array	131 + 0	Higher expression in malignant neoplasms compared to benign.	Kebebew et al. 2005 [60]
Uterine	Protein	EIA, IHC	53 + 30	Higher expression (*p* < 0.05) in patients. No correlation to clinicopathological features.	Ueno et al. 1999 [61]

Abbreviations: AC: Adenocarcinoma; AG array: Angiogenesis array; BCC: Basal cell carcinoma; CSCC: Cutaneous squamous cell carcinoma; CSS: Cancer specific survival; EIA: Enzyme immunoassay; ELISA: Enzyme-linked immunosorbent assay; FACS: Fluorescence activated cell sorting/flow cytometry; HCC: Hepatocellular carcinoma; ICC: Immunocytochemistry; IHC: Immunohistochemistry; ISH: In situ hybridization; LC-MS: Liquid chromatography—mass spectrometry; KA: Keratoacanthoma; LN: Lymph node; LS: Long survival; NR: Not reported; nBCC: Nodular basal cell carcinoma; ns: Not significant; OSCC: Oral squamous cell carcinoma; OTSCC: Oral tongue squamous cell carcinoma; RT-PCR: Real-time or reverse transcription polymerase chain reaction; SCC: Squamous cell carcinoma; SG: Salivary gland; SL: Supraglottic larynx; SS: Short survival; TGF-β1: Transforming growth factor β1; T1 tumor: Tumor extending to mucosal and submucosal layers according to tumor, node, metastasis (TNM) staging for gastric cancer; VEGF-C: vascular endothelial growth factor C; WB: Western blot.

**Table 2:** Expression of matrix metalloproteinase 8 in blood and prognosis.

Cancer.	Plasma/Serum	Method	Study Size (Patients + Healthy Controls)	Expression and Prognosis	Authors
Breast	Serum	Microarray	11 + 10	Higher in patients (*p* = 0.001).	Li et al. 2017 [72]
	Plasma	ELISA	208 + 42	Higher in patients with non-inflammatory breast cancer (*p* = 0.007). Associated with premenopausal status (*p* = 0.06), NPI (*p* = 0.04) and lymph node involvement (pN1-2 *p* = 0.001). Lower levels in patients with risk of distant metastasis (pN3, *p* = 0.003).	Decock et al. 2008 [73]
Colorectal	Serum	IFMA	335 + 47	Higher in patients with advanced disease (Dukes classification *p* < 0.001, T status *p* = 0.004), distant metastasis (*p* < 0.001), tumor in right side of colon (*p* = 0.038). Correlation to worse overall survival (*p* = 0.005)	Böckelman et al. 2018 [74]
	Serum	IFMA	271 + 0	Higher is patients with high mGPS (*p* < 0.001). Negative correlation with tumor-infiltrating mast cells in invasive margin (*p* = 0.005) and tumor centre (*p* = 0.010). Correlation with poor cancer-specific survival (*p* = 0.009).	Sirniö et al. 2018 [75]
	Serum	IFMA	116 + 83	Higher in patients (*p* = 0.0000000015). Correlation with TNM stage (*p* = 0.00045), T status (*p* = 0.0035), distant metastasis (*p* = 0.000054), CLR (*p* = 0.0057), necrosis (*p* = 0.0024), high neutrophil and leukocyte cell count (*p* < 0.05) and peritumoral tumor-destructing inflammatory cell infiltrate (*p* = 0.041).	Väyrynen et al. 2012 [32]
Esophagus	Serum	Microarray	10 + 10	Lower expression in patients (*p* < 0.01).	Tong et al. 2018 [76]
Gastric	Serum	IFMA	233 + 0	Higher expression in patients with intestinal cancer (*p* = 0.044). Patients with intermediate (31–131 ng/mL) serum MMP8 levels had better prognosis (*p* = 0.002).	Laitinen et al. 2018 [35]
Head and neck	Serum	IFMA	33 SCC, 175 benign + 0	Higher in tonsillar SCC patients compared to patients with benign tonsillar disease (*p* = NR).	Ilmarinen et al. 2017 [66]
	Plasma	IFMA	198 + 0	Trend towards favorable outcome (ns.).	Nurmenniemi et al. 2012 [77]
	Plasma	IFMA	136 + 0	Does not correlate with survival or lymph node involvement.	Pradhan-Palikhe et al. 2010 [78]
	Serum	EIA	59 + 0	Higher than healthy persons (*p* < 0.05). Correlation with tumor stage (*p* = NR).	Kuropkat et al. 2004 [79]
	Serum	NR	73 + 74	Higher than healthy persons, correlation with overall TNM status (*p* = NR).	Kuropkat et al. 2002 [80]
Kidney	Serum	ELISA	43+ 10	Surgery lowers MMP-8 levels (*p* = NR).	Kołomecki et al. 2001 [81]
Liver	Serum	IFMA	134 + 0	Worse overall survival (*p* = 0.013). Correlation to BCLC criteria (*p* < 0.0001) and tumor size (*p* < 0.0001).	Lempinen et al. 2013 [82]
Melanoma	Serum	IFMA	117 + 0	Elevated in vascular-invading (*p* = 0.001), ulcerating (*p* = 0.003) and bleeding (*p* = 0.033) melanomas. Correlation to worse outcome (*p* = 0.023).	Vihinen et al. 2008 [83]
Nerves	Plasma	NR	14 + 0	Elevated in carotid body cancer patients (*p* = NR).	Serra et al. 2014 [84]
Pancreas	Serum	MAP kit	109 + 40	Expression higher in cancer patients (*p* = 0.0001).	Park et al. 2012 [85]
Thyroid	Plasma	ELISA	22 + 0	No correlation with tumor presence (*p* = NR).	Komorowski et al. 2002 [86]
Urothelial	Plasma	FACS	135 + 0	No correlation to clinical stage or cancer specific mortality in high-grade tumors.	Svatek et al. 2010 [87]

Abbreviations: BCLC: Barcelona clinic liver cancer criteria; CLR: Crohn’s like lymphoid reaction; FACS: Fluorescence-activated cell sorter; (Fluorokine) MAP kit: Human MultiAnalyte Profiling Base kit, R&D Systems; IFMA: Immunofluorometric assay; IFN-α: Interferon-α therapy; LN: Lymph node; mGPS: Modified Glasgow prognostic score; NPI: Nottingham prognostic index; NR: Not reported; ns.: not significant; TNM: Tumor, node, metastasis staging.

**Table 3:** Matrix metalloproteinase 8 SNPs and prognosis.

Cancer	SNPs	Study Size (Patients + Healthy Controls)	Effect on Patient (*p*-Value)	Authors
Bladder	rs11225395, rs35866072, rs34009635	375 + 375	No effect on bladder cancer risk.	Tsai et al. 2018 [90]
	rs11225395	200 + 200	Reduced risk of bladder cancer (*p* = 0.006).	Srivastava et al. 2013 [91]
	rs1940475	243 invasive, 315 superficial	Protects from invasive phenotype in former smokers (*p* = 0.05).	Kader et al. 2007 [92]
	rs1940475	560 + 560	Trend to increased risk of invasive bladder cancer in never smokers (ns.).	Kader et al. 2006 [93]
Breast	rs11225395, rs35866072, rs34009635	1232 + 1232	No effect on breast cancer risk.	Hsiao et al. 2018 [94]
	rs11225395	6307 + 0	Better overall survival (*p* = 0.021).	Beeghly-Fadiel et al. 2012 [95]
	rs11225395	300 + 300	Not associated with breast cancer risk.	Dȩbniak et al. 2011 [96]
	rs1940475, rs1320632, rs11225395, rs17099436, rs10895353, rs7943404, rs1892886, rs2508383, rs1276284	4470 + 4560	rs1892886: Associated with breast cancer (*p* = 0.0097).	Mavaddat et al. 2009 [97]
	rs10895353, rs7943404, rs11225395, rs1320632, rs1940475, rs1892886, rs17099436, rs2508383, rs1276284	1333 + 0	rs11225395, rs1940475, rs1892886 and rs1276284: Less metastasis (*p* = 0.02, *p* = 0.03, *p* = 0.03, *p* = 0.03, respectively).rs11225395: Higher overall (*p* = 0.02) and disease-specific survival (*p* = 0.02) and less relapse (*p* = 0.04) in early stage patients.	Decock et al. 2007 [98]
Gastric	rs1940475	254 + 0	Higher risk for recurrence (*p* = 0.005) and lower overall survival (*p* = 0.001), recurrence-free survival (*p* = 0.005) and disease-free survival (*p* = 0.011).	Lin et al. 2017 [36]
	rs11225395, rs2155052	79 + 169	No correlation to risk or clinicopathological parameters.	Kubben et al. 2006 [99]
Head and neck	rs11225395, rs35866072, rs34009635	788 + 956	No effect on oral cancer risk.	Hung et al. 2017 [100]
	rs11225395	198 + 0	No significant effect on overall survival in NPC.	Liu et al. 2013 [101]
	rs11225395	136 + 0	No correlation to survival or LNM in HNSCC patients.	Pradhan-Palikhe et al. 2010 [78]
Leukemia	rs11225395, rs35866072, rs34009635	266 + 266	No effect on childhood leukemia risk.	Pei et al. 2017 [102]
Liver	rs11225395	434 + 480	Increased risk of HCC in non-HBV-carriers (*p* = 0.03).	Qiu et al. 2008 [103]
Lung	rs11225395, rs35866072, rs34009635	358 + 716	No effect on lung cancer risk.	Shen et al. 2017 [104]
	rs2155052	501 + 510	Reduced risk for lung cancer (*p* = 0.019), especially in males (*p* = 0.021), ever smokers (*p* = 0.034) and patients with family history of lung cancer (*p* = 0.011). Reduced risk for small cell carcinoma (*p* = 0.023) and squamous cell carcinoma (*p* = 0.008).	González-Arriaga et al. 2008 [105]
Ovarian	rs17099462	417 + 417	Reduced overall survival (*p* = 0.0257).	Wang et al. 2015 [106]
	rs11225395, rs2155052	35 malignant, 51 benign + 37	rs11225395: Increased risk for ovarian cancer (*p* = 0.02) and tendency towards worse overall survival (ns.).	Arechavaleta-Velasco et al. 2014 [107]
Skin	rs1940475	285 SCC, 300 BCC, 218 melanoma + 870	Reduced risk for BCC (*p* = 0.04), no effect in SCC or melanoma.	Nan et al. 2008 [108]
	rs11225395	300 melanoma + 300	Increased risk for melanoma (*p* = 0.017).	Dȩbniak et al. 2011 [96]
Thyroid	rs1940475	31 PTC, 19 FTC and 9 ATC + 0	Present in 80.6% of PTC, 73.6% of FTC and 88.8% of ATC tumors.	Murugan et al. 2011 [109]

Abbreviations: ATC: Anaplastic thyroid carcinoma; BCC: Basal cell carcinoma; FTC: Follicular thyroid carcinoma; HBV: Hepatitis B virus; HCC: Hepatocellular carcinoma: HNSCC: Head and neck squamous cell carcinoma; LNM: Lymph node metastasis; NPC: Nasopharyngeal cancer; ns.: not significant; PTC: Papillary thyroid carcinoma; SCC: Squamous cell carcinoma.

## Abbreviations

DFS	Disease-free survival
ECM	Extracellular matrix
EMT	Epithelial-mesenchymal transition
HNSCC	Head and neck squamous cell carcinoma
MMP	Matrix metalloproteinase
MMP8	Matrix metalloproteinase 8
MMPI	Matrix metalloproteinase inhibitor
OS	Overall survival
OSCC	Oral squamous cell carcinoma
OTSCC	Oral tongue squamous cell carcinoma
PMN	Polymorphonuclear neutrophils
RFS	Recurrence- or relapse-free survival
SCC	Squamous cell carcinoma
SNP	Single nucleotide polymorphism
TGF-β1	Transforming growth factor β1
TIMP-1	Metalloproteinase inhibitor 1
VEGF-C	Vascular endothelial growth factor C
